# Draft Genome Sequence of Collimonas pratensis Strain PMB3(1), an Effective Mineral-Weathering and Chitin-Hydrolyzing Bacterial Strain

**DOI:** 10.1128/MRA.00601-20

**Published:** 2020-09-10

**Authors:** Laura Picard, Philippe Oger, Marie-Pierre Turpault, Stéphane Uroz

**Affiliations:** a Université de Lorraine, INRAe, UMR 1136 Interactions Arbres-Microorganismes, Champenoux, France; b INRAe, UR 1138 Biogéochimie des Écosystèmes Forestiers, Champenoux, France; c Université de Lyon, INSA de Lyon, Université Claude Bernard Lyon 1, CNRS UMR 5240, Lyon, France; University of Maryland School of Medicine

## Abstract

We announce the draft genome sequence of Collimonas pratensis PMB3(1), isolated from the Scleroderma citrinum mycorrhizosphere. In addition to its mineral-weathering effectiveness and antifungal activity, this strain is characterized by genomic features that give it great potential as a biocontrol and plant growth-promoting agent in nutrient-poor soils.

## ANNOUNCEMENT

In temperate regions, forests develop mostly on nutrient-poor and acidic soils, in which minerals represent essential sources of nutritive cations (Ca, Mg, K, Fe). Cations entrapped in these minerals are made bioavailable to the plants through a dissolution process termed “mineral weathering,” in which bacteria play an essential part (1). *Collimonas* species are considered very effective mineral-weathering and plant-growth-promoting bacteria (1, 2). Representatives of this genus belong to the *Oxalobacteraceae* family, which comprises four described species (*C. anthrihumi*, *C. arenae*, *C. fungivorans*, and *C. pratensis*) (3–5). Collimonads are members of the rare biosphere found in acidic soils, with low nutrient availability and often in interaction with fungi (6–9). Strain PMB3(1) was isolated from oak rhizosphere on 10% tryptic soy agar (TSA) medium and cryopreserved in 40% glycerol.

Strain PMB3(1) was grown at 25°C in LB medium to late exponential phase. The cell pellet was lysed with lysozyme (1 mg/ml) at 50°C for 1 h, sodium dodecyl sulfate (final concentration, 1%), and proteinase K (final concentration, 1 mg/ml), followed by chloroform purification and ethanol precipitation as described by Pospiech and Neumann (10). The libraries were prepared using the Kapa HyperPlus kit (Roche) and Nextera XT DNA library preparation kit (Illumina), following the manufacturer’s instructions. A combination of a mate pair library of 3 kb, an unpaired fragment library of 500-bp fragments done on a GS-FLX 454 system (Roche), and an Illumina library of 100-bp reads done on a MiSeq instrument (Beckman Coulter Genomics, Danvers, MA, USA) was effected. A total of 111,000 454 reads and 41 million Illumina reads were generated, providing 309 Mb of 454 reads and 4,153 Mb of Illumina reads.

For all of the following programs, default parameters were used except where otherwise specified.

*De novo* assembly was performed using MIRA (version 4.0) (11). The draft genome has 137 contigs larger than 500 bp, which were assembled into 16 scaffolds, with a calculated total length of 5,613,242 bp (average depth of coverage, 30×), a G+C content of 59%, and an *N*_50_ contig size of 380,062 bp. The largest scaffold generated was 1,367,376 bp, and the *N*_50_ scaffold size was 559,889 bp. Genome-based taxonomy assigned strain PMB3(1) to the species Collimonas pratensis (digital DNA-DNA hybridization [dDDH] value, 84%; https://tygs.dsmz.de). The genome of PMB3(1) comprises a total of 5,136 predicted protein-coding genes and 52 tRNA genes.

According to RAST (12), 12% of the predicted proteins appeared involved in carbohydrate metabolism, including the Entner-Doudoroff pathway, in which glucose is oxidized to gluconate. The production of gluconate was proposed to play a role in mineral weathering (13). However, no pyrroquinoline quinone (PQQ) system was detected. Based on genome analysis, strain PMB3(1) may produce metabolites with antibacterial (rhizomide and feglymycin) or antifungal (iturin) activities as well as siderophores and organic acids. These activities suggest that PMB3(1) is well equipped to live in the rhizosphere of plants growing in nutrient-poor environments, to inhibit fungal growth, and to mobilize nutrients through its mineral-weathering potential, making it a promising growth-promoting and biocontrol agent (7, 14).

### Data availability.

The whole-genome and raw sequences are available under the accession no. WXXL00000000 and SRX8380211 through SRX8380213, respectively.
